# An Open-Label, Single-Arm, Multicentric, Prospective, Phase IV Study to Evaluate the Safety and Effectiveness of Indacaterol/Mometasone/Glycopyrronium Dry Powder Inhaler (DPI) in the Management of Asthma Patients (OASIS Study)

**DOI:** 10.7759/cureus.100370

**Published:** 2025-12-29

**Authors:** Saurabh Karmakar, Suresh G Bhate, Vijaykumar B Barge, Vinod K Kumar, Anjali R Nath, Ekta Sinha, Sagar Bhagat, Saiprasad Patil, Sumit Bhushan, Rujuta Gadkari, Hanmant Barkate

**Affiliations:** 1 Department of Pulmonary Medicine, All India Institute of Medical Sciences, Patna, IND; 2 Department of Pulmonary Medicine, Jeevan Rekha Hospital, Belgaum, IND; 3 Department of Medicine, Chhatrapati Pramilaraje Hospital, Kolhapur, IND; 4 Department of Pulmonary Medicine, New Leelamani Hospital Pvt. Ltd., Kanpur, IND; 5 Department of Pulmonary Medicine, Citizen Hospital, Bengaluru, IND; 6 Pulmonology, Glenmark Pharmaceuticals Limited, Mumbai, IND; 7 Global Medical Affairs, Glenmark Pharmaceuticals Limited, Mumbai, IND; 8 Global Medial Affairs, Glenmark Pharmaceuticals Limited, Mumbai, IND

**Keywords:** asthma, dry powder inhaler, glycopyrronium, indacaterol, mometasone

## Abstract

Background: Asthma is a global health concern that requires an effective management approach. The OASIS study aims to evaluate the safety and effectiveness of the fixed-dose combination (FDC) of indacaterol acetate, mometasone furoate, and glycopyrronium bromide (IND/MF/GLY) delivered once daily via a dry powder inhaler (DPI) in Indian adults with inadequately controlled asthma, notwithstanding the global evidence supporting its use.

Methods: This 12-week, open-label, single-arm, multicenter phase IV study was conducted on asthmatic patients at five sites in India (All India Institute of Medical Sciences (AIIMS), Bihar; Jeevan Rekha Hospital, Karnataka; Rajarshee Chhatrapati Shahu Maharaj Government Medical College and Chhatrapati Pramila Raje Hospital, Maharashtra; New Leelamani Hospital Pvt Ltd, Kanpur; and Citizen Hospital, Karnataka). The study was approved by the Institutional Ethics Committee of AIIMS (approval no. AIIMS/Pat/EC/2024/1197) and registered with Clinical Trials Registry - India (CTRI: 2023/12/060605). The primary and secondary endpoints were safety and effectiveness, respectively. Effectiveness included changes in lung function, Asthma Control Questionnaire-5 (ACQ-5) score, rescue medication use, treatment satisfaction, and adherence.

Results: A total of 182 patients were enrolled in the study, of which 180 (mean age 46.2 ± 12.9 years; 59.9% male) completed the study. Over 12 weeks, treatment-emergent adverse events were minimal (2.7%) with no serious adverse events. Significant improvements were observed in lung function at both week 4 (forced expiratory volume in 1sec (FEV₁): +264.5 ± 53.5 mL; forced vital capacity (FVC): +225.6 ± 18.4 mL) and week 12 (FEV₁: +458.2 ± 66.2 mL; FVC: +522.9 ± 271.8 mL), alongside ACQ-5 score reductions (week 4: −0.71 ± 0.07; week 12: −1.45 ± 0.03). Rescue medication use declined, and adherence remained high (98.4%), with strong patient and physician satisfaction.

Conclusion: Once-daily IND/MF/GLY FDC DPI demonstrated a favorable safety profile and significant improvement in asthma control in Indian adults.

## Introduction

Despite advancements in asthma management, inadequately controlled asthma affects 262 million people globally and 35 million cases are reported in India, causing 455,000 deaths annually worldwide [1-3]. According to the Global Burden of Disease (GBD) 2021 Report, India contributes to 46% of global asthma deaths, an increase from 43% reported in 2019 [4].

The 2025 GINA guidelines reaffirm the importance of achieving optimal asthma control by managing current symptoms and minimizing future exacerbation risks. For adults and adolescents (≥18 years) with uncontrolled asthma, adding a long-acting muscarinic antagonist (LAMA) is recommended despite ongoing treatment with medium- or high-dose inhaled corticosteroids (ICSs) and a long-acting β₂-agonist (LABA) [5].

The indacaterol acetate, mometasone furoate, and glycopyrronium bromide (IND/MF/GLY) fixed-dose combination (FDC) targets distinct pathways in asthma pathophysiology. Indacaterol (Ultra LABA), Mometasone (potent ICS), and Glycopyrronium (Ultra LAMA) work synergistically to enhance airway function and reduce inflammation, offering a comprehensive approach to asthma management [6]. Studies have shown that single-inhaler triple therapy (SITT) improves compliance by reducing treatment complexity, leading to higher adherence rates and better persistence [7-9]. Moreover, the convenience of delivering this combination through a single inhaler enhances treatment adherence while simultaneously reducing costs, effectively addressing a critical barrier in asthma management [10].

FDC of medium-dose ICS regimen of IND/MF/GLY (150/160/50 mcg) once-daily dry powder inhaler (DPI) has been evaluated in multiple large-scale studies globally as maintenance therapy of inadequately controlled asthma in adults [11,12]. However, there is no documented study in the Indian population available to determine the safety and effectiveness of the combination for managing inadequately controlled asthma in adults.

The OASIS study aims to evaluate the safety and effectiveness of an FDC of IND/MF/GLY delivered via a DPI in adult Indian patients with uncontrolled asthma receiving maintenance therapy, with effectiveness assessed using lung function (FEV₁), asthma control (ACQ-5 score), and treatment adherence, and safety evaluated through the incidence of adverse events.

## Materials and methods

Study design

This was an open-label, single-arm, multicentric, prospective phase IV study evaluating the safety and effectiveness of the DPI combination of IND (150 mcg), MF (160 mcg), and GLY (50 mcg) once a day in patients with uncontrolled or severe asthma. This study was conducted over 12 weeks across five centers in India (All India Institute of Medical Sciences (AIIMS), Bihar; Jeevan Rekha Hospital, Karnataka; Rajarshee Chhatrapati Shahu Maharaj Government Medical College and Chhatrapati Pramila Raje Hospital, Maharashtra; New Leelamani Hospital Pvt Ltd, Kanpur; and Citizen Hospital, Karnataka). It adheres to the International Council for Harmonization (ICH) Good Clinical Practice (GCP) guidelines [13], the Declaration of Helsinki 2024 [14], and New Drugs and Clinical Trials (NDCT) 2019 Rules [15]. Approval from the Institutional Ethics Committee at all the study sites was obtained prior to the enrollment of the first patient (no. AIIMS/Pat/EC/2024/1197). In addition, written informed consent was obtained from all study participants before their inclusion in the study. The study was registered with Clinical Trials Registry - India (CTRI: 2023/12/060605).

Participants

Eligible patients included adults aged 18 years or older with a confirmed asthma diagnosis and symptomatic on maintenance therapy with ICS + LABA or ICS + Formoterol, with an Asthma Control Questionnaire (ACQ-5) score ≥1.5 at baseline [16], and at least one asthma exacerbation in the previous year. Exclusion criteria were patients requiring hospitalization for life-threatening conditions, acute exacerbation, smoking history >10 pack-years, prior triple therapy (ICS/LABA/LAMA), hypersensitivity to study drugs, and specific risks for women of childbearing potential (WOCBP).

Outcome measures

The primary outcome measure was to evaluate the safety of IND/MF/GLY DPI in the management of asthma. The secondary outcome measures included assessing its effectiveness in the Indian population, in terms of improvement of lung function as measured by changes in trough FEV₁ and FVC, and asthma control as measured by changes and percentage changes in ACQ-5 scores.

Statistical analysis

Descriptive statistics summarized the demographic and baseline characteristics for all analysis sets. Continuous variables were presented as the number of participants, mean, standard deviation (SD), minimum, median, and maximum, while categorical variables were reported as frequencies and percentages. Safety data, including adverse events, were analyzed by system organ class, preferred term, frequency, and severity. Skewed data were presented as medians and ranges. The student's t-test was applied to compare continuous variables with a normal distribution, while the Wilcoxon signed-rank test was employed for skewed data, with adjusted p-values. A p-value of <0.05 was considered statistically significant. All statistical analyses were conducted using R software (version 4.3.2).

Determination of sample size

For a 12-week treatment period, assuming the 5.5% rate of drug-related AEs (similar to the ARGON study), a single-group design was used to obtain a two-sided 95% confidence interval for a single proportion. The sample proportion was assumed to be 5.5%. To produce a confidence interval with a width of no more than 0.07, 163 subjects were needed. Anticipating a 10% dropout rate, 182 subjects were enrolled. The sample size was computed using PASS 2022, version 22.0.2 (NCSS, LLC).

## Results

**Figure 1:**
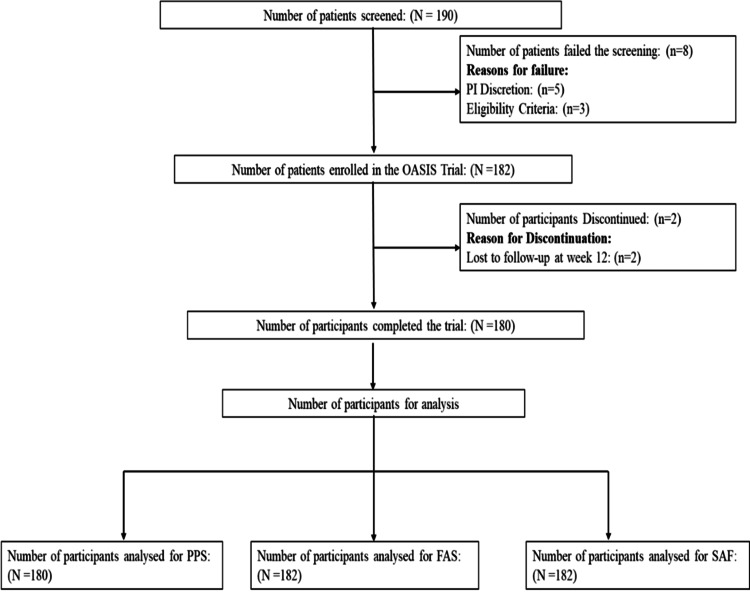
Flowchart from patient screening to data analysis.

In this study, 190 participants were screened for eligibility. A total of 182 participants were enrolled, out of which 180 completed the study (Figure 1). For data analysis, 180 participants were included in the per-protocol set (PPS), while 182 participants were considered for the full analysis set (FAS) and safety (SAF) outcome.

In this study, the mean (± SD) age of the participants was 46.21 ± 12.95 years, predominantly consisting of male participants (59.89%). The baseline assessments indicated inadequate asthma control, reflected by an average ACQ-5 score of 3.15 ± 0.52 and a mean of 1.17 ± 0.38 exacerbation episodes reported in the previous year. For details about demographic and baseline characteristics, refer to Table 1.

**Table 1:** Demographic and baseline characteristics. ACQ, Asthma Control Questionnaire [16]; FEV1, forced expiratory volume in one second; FVC, forced vital capacity; n, number of participants; N, total number of participants; %, percentage of participants; SD, standard deviation; ICS-LABA, fixed-dose combination of inhaled corticosteroid-long-acting β₂-agonist.

Parameter	(N = 182) Mean ± SD
Age (years)	46.21 ± 12.95
Gender, n (%)	
Male	109 (59.89%)
Female	73 (40.10%)
Height (cm)	165.46 ± 5.98
Weight (kg)	63.89 ± 7.61
Body mass index (BMI) (kg/m²)	23.29 ± 2.55
Baseline FEV1 (ml)	1252.25 ± 443.34
Predicated FEV1 (%)	52.44 ± 11.42
Baseline FVC (ml)	2218.90 ± 681.88
Baseline ACQ-5 score	3.15 ± 0.52
Duration of asthma (years)	3.14 ± 1.98
Mean number of episodes of Exacerbation experienced in the past year	1.17 ± 0.38
Comorbidities, n (%)	
Hypertension	23 (12.63%)
Diabetes mellitus	20 (10.99%)
Hypothyroidism	8 (4.40%)
Prior medications for asthma, n (%)	
ICS-LABA	182 (100%)
Mometasone and formoterol	24 (13.18)
Budesonide and formoterol (SMART)	83 (45.6)
Beclometasone and formoterol	1 (0.54)
Fluticasone and formoterol	2 (1.09)
Salmeterol and formoterol	72 (39.56)

Safety endpoints

A total of 28 treatment-emergent adverse events (TEAEs) (15.38%) were reported, primarily in the respiratory system and general disorders, the most common being cough, with seven events (3.85%), and fever, with five events (2.75%). Notably, there were no serious TEAEs (STEAEs) recorded. In addition, only five events (2.74%) of the TEAEs were classified as drug-related, including cough (3.85%), wheezing (0.55%), and upper respiratory tract infections URTI (0.55%) (Table 2).

**Table 2:** Overall adverse events in the study participants AE, number of adverse events reported; TEAE, treatment-emergent adverse events; URTI, upper respiratory tract infection

Parameter	Adverse events	No. of AEs reported (N = 182) E (%)
Treatment-emergent adverse events (TEAEs)	-	28 (15.38)
General disorders and administration site conditions	Fever	05 (2.75)
	Cold	03 (1.65)
	Body ache	01 (0.55)
	Body pain	02 (1.10)
Respiratory, thoracic, and mediastinal disorders	Cough	07 (3.85)
	Stuffy nose	01 (0.55)
	URTI	01 (0.55)
	Wheezing	01 (0.55)
Musculoskeletal and connective tissue disorders	Back pain	01 (0.55)
	Muscle cramp	01 (0.55)
Nervous system disorders	Headache	03 (1.65)
Gastrointestinal disorders	Abdominal Bloating	01 (0.55)
	Diarrhea	01 (0.55)
Drug-related TEAEs	-	05 (2.74)
Respiratory, thoracic, and mediastinal disorders	Cough	03 (1.65)
	URTI	01 (0.55)
	Wheezing	01 (0.55)
Any serious TEAEs (STEAEs)	-	00 (00)

Effectiveness endpoints

**Figure 2:**
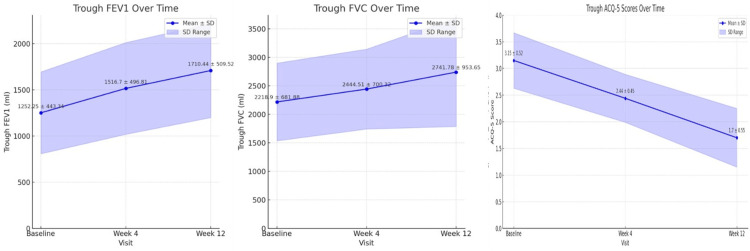
Change in mean trough FEV1, trough FVC, and ACQ-5 over study duration and changes in FEV1, FVC, and ACQ-5 from baseline FEV1, forced expiratory volume in one second; FVC, forced vital capacity; ACQ, Asthma Control Questionnaire [16] Images created by the authors with Microsoft PowerPoint (Microsoft Corp., USA)

The effectiveness endpoints indicated significant improvements in lung function and asthma control among participants. At baseline, the mean (±SD) mL, trough FEV1 was 1252.25 (±443.34), which increased to 1516.70 (±496.81) at week 4 and further to 1710.44 (±509.52) at week 12, demonstrating a significant increase of 264.45 (±53.47) and 458.19 (±66.18), respectively (both p < 0.001). Similarly, the mean (±SD) mL, trough FVC increased from 2218.90 (±681.88) at baseline to 2444.51 (±700.32) at week 4 and 2741.78 (±953.65) at week 12, with significant increases of 225.61 (±18.44) and 522.88 (±271.77), respectively (both p < 0.001). In addition, the ACQ-5 score exhibited a reduction from 3.15 (±0.52) at baseline to 2.44 (±) 0.45 at week 4 and 1.70 (±) 0.55 at week 12, reflecting a significant reduction of -0.71 (±) 0.07 and -1.45 (±) 0.03, respectively (both p < 0.001) (Figure 2)

Other endpoints

The study reported improvements in asthma control among the participants, evidenced by a mean percentage change in ACQ-5 scores of 22.53% at week 4 and 46.03% at week 12.

Mild exacerbation was reported by eight (4.39%) and five (2.77%) subjects at weeks 4 and 12, respectively. No moderate or severe exacerbations were experienced throughout the study duration.

The average use of rescue medication (Salbutamol) exhibited a decrease, with 7.69% (n = 14) of patients utilizing it during weeks 0-4 compared to 7.22% (n = 13) in weeks 4-12. Compliance among the participants was 98.39% (four weeks) and 98.42% (12 weeks).

**Figure 3:**
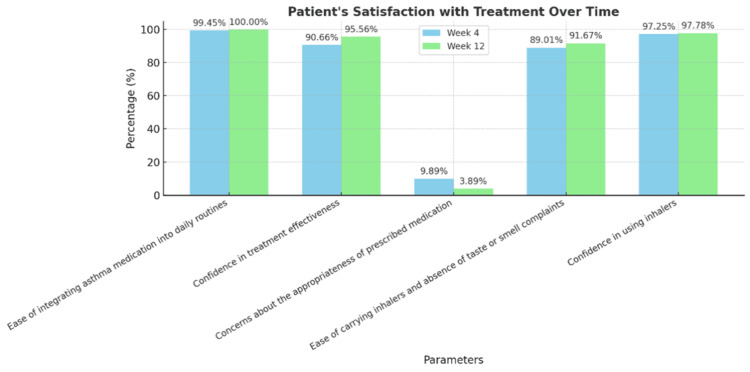
Assessment of patients' satisfaction with the treatment Image created by the authors with Microsoft PowerPoint (Microsoft Corp., USA)

**Figure 4:**
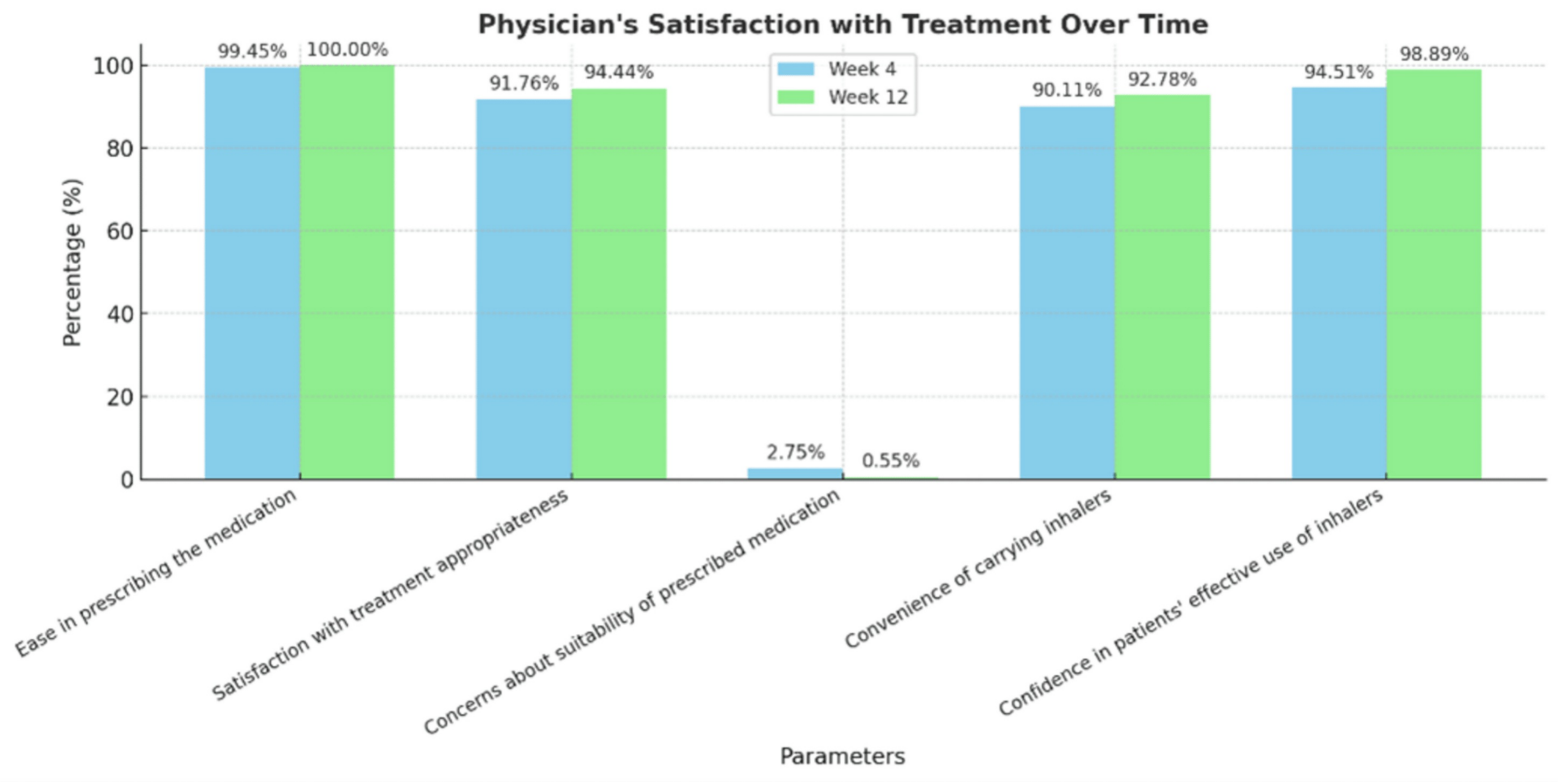
Assessment of physicians' satisfaction with the treatment Image created by the authors with Microsoft PowerPoint (Microsoft Corp., USA)

Participant and physician satisfaction with asthma treatment improved significantly over the 12-week period. The participants reported greater ease in integrating medication into daily routines, increased confidence in treatment effectiveness, and fewer concerns about medication appropriateness (Figure 3). Physicians also experienced higher satisfaction, with enhanced confidence in prescribing the medication, improved perceptions of treatment suitability, and greater confidence in patients’ ability to effectively use their inhalers (Figure 4).

## Discussion

The OASIS study assessed the safety and effectiveness of an FDC of IND/MF/GLY via DPI for inadequately controlled asthma. It showed a strong safety profile, low adverse event rates, and significant clinical benefits, including improved FEV1, FVC, reduced asthma symptoms and exacerbations, and high patient and physician satisfaction. 

Safety findings of this study were consistent with other studies like the ARGON and IRIDIUM, which reported 5.5% and 9.2% of drug-related TEAEs, respectively. In addition, in this study, pneumonia or any other SAEs was not observed, highlighting the favorable safety profile of IND/MF/GLY in the study patient population. In comparison, in the ARGON and IRIDIUM studies, pneumonia was identified as a notable serious adverse event, reported in 1.1% (n = 5) and 0.5% (n = 3) of patients receiving high-dose IND/MF/GLY, respectively [11,12]. Pneumonia and other serious adverse events were reported across the ARGON, IRIDIUM, and CAPTAIN trials [11,12,17]. However, in the CAPTAIN and IRIDIUM trials, no significant difference in pneumonia incidence was observed between high- and medium-dose ICS regimens. These findings align with meta-analyses in asthma that found no consistent increase in pneumonia risk associated with ICS use, even at higher doses [18,19]. Across the ARGON, IRIDIUM, and CAPTAIN trials, the most commonly reported adverse events included asthma exacerbations, naso-pharyngitis, URTIs, headache, and bronchitis, with varying frequencies [11,12,17].

The OASIS study supports the favorable and consistent safety profile of IND/MF/GLY DPI, with lower incidence of both TEAEs and drug-related AEs, and no observed SAEs or pneumonia, compared to previous trials like ARGON, IRIDIUM, and CAPTAIN. This positions IND/MF/GLY as a well-tolerated treatment option in asthma management.

The improvement of lung function (FEV1 and FVC) reported in this study was comparable to that reported in ARGON and IRIDIUM studies. IND/MF/GLY DPI demonstrated a significant improvement in mean trough FEV1 (458.19 ± 66.18) at 12 weeks, similar to the ARGON study in which high-dose IND/MF/GLY improved trough FEV1 by 334 ml at week 24. Similar insights were received from the IRIDIUM trial in which improvement in trough FEV1 for medium and high dose IND/MF/GLY at week 26 was 299 ml and 320 ml, respectively, and was significantly superior compared to both MF/IND (high and medium doses) and high dose of FLU/SAL (p < 0.001) [11,12]. The CAPTAIN trial also showed huge improvements in FEV1 of 134 (FF 100 mcg/Vi 25 mcg/Umec 62.5 mcg) and 168 ml (FF 200 mcg/Vi 25 mcg/Umec 62.5 mcg) from baseline. This was significantly better compared to FF/Vi (100/25 mcg and 200/25 mcg) (p < 0.001), respectively [17]. Collectively, these results indicate that triple inhaler therapies offer substantial and consistent benefits in improving lung function in patients with asthma.

The average use of rescue medication can be considered a surrogate for asthma control [20]. Similar to our study, a post-hoc analysis of the IRIDIUM study reported a decrease in use of rescue medication in the high-dose IND/GLY/MF group (−0.12 (−0.27 to 0.03)) compared to high-dose SAL/FLU [21]. In the CAPTAIN trial, the specific numbers for rescue medication use showed a clear difference between the treatment groups. Patients on the triple therapy (fluticasone furoate/umeclidinium/vilanterol) had a mean reduction of 0.5 puffs per day compared to those on dual therapy (fluticasone furoate/vilanterol) [20]. Less use of rescue medications, especially SABAs, indicates better asthma control and reduced disease severity. It reflects improved adherence to controller therapies like ICS, leading to fewer exacerbations, lower healthcare utilization, and better overall patient outcomes [22,23].

 In this study ACQ-5 score showed a significant decline, further underscoring the effectiveness of IND/MF/GLY DPI in achieving better asthma control. Similarly, in the ARGON study, both the high- and medium-dose IND/GLY/MF significantly improved the ACQ-7, with the high-dose showing a treatment difference of -0.124 (95% CI: -0.216, -0.032) (p = 0.004), indicating notable improvement in asthma control [11]. Likewise, in the CAPTAIN trial, improvements in ACQ-7 scores were seen across all treatment groups, with mean changes exceeding the minimal clinically important difference of 0.5 points, which suggests enhanced asthma control [18]. A network metanalysis of five phase-III RCTs of triple combination therapy reported comparable improvement of ACQ scores in which both MD and HD ICS/LABA/LAMA FDCs and HD ICS/LABA FDC+TIO were equally (p > 0.05) effective in improving ACQ score, although a trend toward significance (p = 0.05) was detected for HD ICS/LABA/LAMA versus MD ICS/LABA/LAMA FDC [24]. Both medium- and high-dose regimens consistently showed clinically meaningful improvements, supporting their role in optimal asthma management.

Non-compliance with double or multiple inhaler triple therapy in asthma patients can lead to suboptimal treatment outcomes, including increased asthma exacerbations, reduced lung function, and a higher risk of hospitalization [7]. In this study, the compliance among participants with the FDC of IND/MF/GLY DPI was found to be notably high (98.39% at week 4 and 98.42% by week 12), indicating good treatment adherence. This observation aligns with findings from a retrospective cohort study that evaluated adherence, persistence, and effectiveness among combination therapy (ICS and LABA in one inhaler) versus concurrent therapy (ICS and LABA in separate inhalers). Patients on combination therapy demonstrated significantly better adherence, filling 0.9 more prescriptions annually, and higher persistence, with a 17% lower likelihood of treatment discontinuation. Improved adherence and persistence in combination therapy users were associated with a 17% reduced risk of moderate to severe asthma exacerbations, emphasizing the advantages of simplified regimens for asthma management [25]. These findings collectively highlight the importance of the role of simplified FDC regimens in enhancing adherence, reducing treatment discontinuation, and improving asthma control.

Patient satisfaction is a key factor in asthma management, as it promotes better treatment adherence, improved symptom control, and enhanced quality of life. Conversely, dissatisfaction can lead to poor adherence, worsening asthma control, and further reducing satisfaction [26]. These results affirm IND/MF/GLY as an effective, well-tolerated therapy with potential to improve asthma control and satisfaction.

The multicentric design of this study ensured a diverse patient population, enhancing its real-world relevance, while the open-label format promoted greater patient involvement and compliance. However, the OASIS study has several limitations. The single-arm, open-label design without a comparator group limits causal inference and precludes direct assessment of relative effectiveness compared with standard therapies. The relatively short follow-up duration of 12 weeks restricts the evaluation of long-term safety and sustained efficacy. Treatment adherence was assessed using self-reported measures, which may introduce reporting bias. In addition, the absence of subgroup analyses based on baseline disease severity, age, sex, or comorbidities limits the interpretation of differential treatment responses. Notably, the greater improvement observed in Indian patients compared with other studies may reflect differences in baseline disease characteristics, genetic or environmental factors, or adherence patterns; however, these hypotheses require further investigation in controlled, long-term studies.

## Conclusions

The OASIS study suggests that IND/MF/GLY DPI is generally safe and well-tolerated in Indian adults with inadequately controlled asthma, with a low incidence of treatment-emergent adverse events and no reported serious adverse events. Significant improvements in lung function (trough FEV1, FVC), asthma symptoms, and exacerbation rates, along with decreased ACQ-5 scores, demonstrate its therapeutic effectiveness. High adherence rates and positive feedback from patients and physicians further emphasize its potential to enhance treatment outcomes.
